# Nutritional strategies for Nile tilapia: protein and carbohydrate balances in saline-alkaline aquaculture

**DOI:** 10.1186/s40104-025-01215-8

**Published:** 2025-06-17

**Authors:** Wei Liu, Erchao Li, Chang Xu, Liqiao Chen, Xiaodan Wang

**Affiliations:** 1 School of Life Sciences, East China Normal University, Shanghai, 200241 China; 2 Key Laboratory of Tropical Hydrobiology and Biotechnology of Hainan Province, Hainan Aquaculture Breeding Engineering Research Center, School of Marine Biology and Aquaculture, Hainan University, Haikou, 570228 China

**Keywords:** Glucose metabolism, Gut microbiota, *Oreochromis niloticus*, Protein-to-carbohydrate ratio, Salinity-alkalinity stress, Transcriptomics

## Abstract

**Background:**

The development of saline-alkaline water aquaculture is an important part of the development of the global food supply. However, there is still limited knowledge about nutritional strategies for aquaculture in saline-alkaline water, including essential nutrients such as proteins and carbohydrates. In this study, our objective was to elucidate the role of different protein-to-carbohydrate ratios in the adaptation of Nile tilapia (*Oreochromis niloticus*) to salinity-alkalinity stress.

**Results:**

Fish were fed three isoenergetic (16.5 kJ/g) and isolipidic (60 g/kg) diets with different protein-to-carbohydrate ratios (27% protein and 35% carbohydrate; 35% protein and 25% carbohydrate; 42% protein and 15% carbohydrate) for 50 d. Nile tilapia (0.44 ± 0.03 g) were exposed to both freshwater (salinity: 0.2 PSU; alkalinity: 0.5 g/L NaHCO₃) and saline-alkaline water (salinity: 16.0 PSU; alkalinity: 3.0 g/L NaHCO₃) to observe changes in growth performance, whole-body composition, and antioxidant capacity. To further elucidate the role of protein-to-carbohydrate ratios, we performed gut microbiota and transcriptomic analyses. The results revealed that salinity-alkalinity stress induced oxidative stress, damaged the gill tissue structure, caused hepatocyte cytoplasmic vacuolation, increased the energy demand and the abundance of intestinal pathogens, and ultimately inhibited the growth of tilapia. A diet containing 27% protein and 35% carbohydrate significantly alleviated oxidative stress in tilapia, increased their crude protein content, and ultimately significantly improved the growth performance. Further analyses of the intestinal microbiota and transcriptomics revealed that a diet containing 27% protein and 35% carbohydrate increased the abundance of probiotics in the gut and upregulated energy metabolism pathways related to glucose metabolism.

**Conclusions:**

The diet containing 27% protein and 35% carbohydrate increased the abundance of probiotics in the gut, promoted energy metabolism, and improved the growth performance of tilapia under long-term salinity-alkalinity stress. This study evaluated the impact of protein and carbohydrate levels on the growth of tilapia in saline-alkaline water, offering theoretical support for the development of the saline-alkaline water feed industry. This study also highlighted the crucial role of saline-alkaline water aquaculture in global food security and nutritional supply.

## Introduction

With the deterioration of global salinization and increasing demand for aquatic food worldwide, saline-alkaline water aquaculture urgently needs development [1–3]. It not only is beneficial for the utilization of global water resources but also supplies more food for the growing population [4]. Saline-alkaline water is characterized by high salinity, high alkalinity, high pH and complex ion composition, so salinity-alkalinity stress seriously affects the survival, growth and reproduction of aquatic animals, ultimately leading to a reduction in biomass and species abundance in saline-alkaline waters [5–7]. Therefore, developing appropriate methods to improve the adaptability of aquatic animals under the salinity-alkalinity stress is critical for the development of saline-alkaline aquaculture [8].

Saline-alkaline conditions are known to induce a variety of physiological stresses, including osmotic imbalance, alkalosis, and ammonia toxicity [5, 6, 9]. Moreover, these conditions compel fish to expend substantial energy to maintain osmotic balance, often resulting in inhibited growth and development [10]. For example, studies on medaka fish (*Oryzias latipes*) have revealed significant upregulation of genes associated with energy and ion regulation under alkaline stress, whereas genes involved in immune and reproductive functions are markedly suppressed [11]. Additionally, the research indicates that increased alkalinity can alter the intestinal microbiota in *Cyprinus carpio *Songpu, leading to a decrease in probiotic populations and adversely impacting growth and energy efficiency [12]. Many studies have shown that fish need more energy under salinity-alkalinity stress [13–16].

Protein is an essential nutrient for the growth of fish [17], and protein catabolism is an important source of energy for aquatic animals [18]. Generally, the catabolism of protein causes the blood ammonia level of fish to rise [19]. Salinity-alkalinity stress has been demonstrated to inhibit the excretion of blood ammonia, resulting in ammonia toxicity in fish [20]. Conversely, as a direct energy source, carbohydrates play a crucial role in the stress response and have a significant protein-sparing effect [21]. In addition, studies have shown that carbohydrates are the main energy source for fish to cope with salinity stress [22, 23]. Therefore, whether adjusting the ratio of protein-to-carbohydrate (N:C) in diets can reduce the burden of ammonia metabolism of fish under salinity-alkalinity stress and increase the energy supply, thus supporting the osmotic regulation of fish in saline-alkaline waters, is a topic worthy of further study.

Nile tilapia (*Oreochromis niloticus*) are widely farmed around the world because of their good stress resistance and high economic value [24, 25]. As an omnivorous fish, Nile tilapia also have good dietary carbohydrate utilization capabilities [26]. Previous studies have shown that the protein level in the diet is between 32% and 38% for juvenile Nile tilapia [27, 28], and tilapia can tolerate 32%–36% carbohydrate levels without affecting growth performance [29, 30]. Therefore, Nile tilapia is a good fish model for metabolic research under the salinity-alkalinity stress. This research was carried out to explore how different protein-to-carbohydrate ratios affect their growth performance, antioxidant activities, carbohydrate metabolism, transcriptomic responses, and gut microbiota composition. The findings of this study aimed to elucidate the mechanisms of saline-alkali tolerance in Nile tilapia and provide a scientific basis for enhancing aquaculture practices in saline-alkaline environments.

## Materials and methods

### Diets, animals and experimental design

Three isoenergetic (16.5 kJ/g) and isolipidic (60 g/kg) diets were formulated with varying N:C ratios: LP (27% protein, 35% carbohydrate), MP (35% protein, 25% carbohydrate), and HP (42% protein, 15% carbohydrate). According to previous research, corn starch served as the primary carbohydrate source, whereas soybean meal and corn meal were the main protein sources [31, 32]. The compositions of the 3 experimental diets were presented in Table 1. All powder ingredients were sieved twice with a 60-mesh strainer and then mixed thoroughly following the formula. An F-26 II machine (SCUT Industrial Factory, Guangdong, China) was subsequently used to process particles with a diameter of 2 mm. After being air-dried at room temperature, the diets were stored at −20 °C until use.

**Table 1:** Formulation and chemical composition of the experimental diets (dry matter basis)

Item	Diets		
Ingredients, g/kg	P27/C35	P35/C25	P42/C15
Fish meal	80	80	80
Soybean meal	210	290	370
Corn meal	180	230	280
Corn oil	44	41	38
Corn starch	350	250	150
Cellulose	54	27	0
Vitamin mix^1^	20	20	20
Mineral mix^2^	20	20	20
Ca(H_2_PO_4_)_2_	10	10	10
Choline chloride	2	2	2
Carboxy methyl cellulose	30	30	30
Total	1,000	1,000	1,000
Chemical composition, %			
Moisture	8.96	9.35	11.09
Crude protein	26.91	34.75	41.65
Crude lipid	6.10	5.93	6.08
Ash	6.31	7.09	7.61
Total energy, kJ/g^3^	14.78	14.84	14.81

^1^Vitamin premix (mg/kg): vitamin A (500,000 IU/g), 8 mg; vitamin D_3_ (1,000,000 IU/g), 2 mg; vitamin K, 10 mg; vitamin E, 200 mg; thiamine, 10 mg; riboflflavin, 12 mg; pyridoxine, 10 mg; calcium pantothenate, 32 mg; nicotinic acid, 80 mg; folic acid, 2 mg; vitamin B_12_, 0.01 mg; biotin, 0.2 mg; choline chloride, 400 mg; vitamin C-2- polyphosphate (150 mg/g vitamin C activity), 400 mg

^2^Mineral premix (mg/kg): zinc (ZnSO_4_·7H_2_O), 50.0 mg; iron (FeSO_4_·7H_2_O), 40 mg; manganese (MnSO_4_·7H_2_O), 15.3 mg; copper (CuCl_2_), 3.8 mg; iodine (KI), 5 mg; cobalt (CoCl_2_·6H_2_O), 0.05 mg; selenium (Na_2_SeO_3_), 0.09 mg

^3^Calculated values based on 23.6, 39.5 and 17.2 kJ/g for protein, lipid and nitrogen-free extract, respectively [33]

Juvenile Nile tilapia were obtained from Changsheng Fish Farm (Hainan, China) and acclimatized in four 600-L tanks at 28 ± 2 °C. During the final 6 d of the acclimation period, the salinity and alkalinity in two randomly selected tanks were gradually increased to 16.0 g/L and 3.0 g/L NaHCO_3_, respectively, through daily water changes (salinity rose 2–3 PSU and alkalinity rose 0.5 g/L NaHCO_3_ per day). During the acclimatization period, the fish were fed the commercial diet produced by Tongwei Company (Chengdu, China). After acclimation, 225 fish (0.44 ± 0.03 g) from freshwater (FW; salinity: 0.2 PSU; alkalinity: 0.5 g/L NaHCO_3_) and another 225 healthy fish (0.44 ± 0.03 g) from saline-alkaline water (SW; salinity: 16.0 PSU; alkalinity: 3.0 g/L NaHCO_3_) were randomly divided into 18 experimental tanks (30 cm × 60 cm × 35 cm). The nine freshwater tanks and nine saline-alkaline tanks were randomly assigned to three experimental groups, each comprising three replicates. The trial lasted for 50 d. Throughout the feeding trial, tilapia were fed twice daily at 08:00 and 16:00 to apparent satiation, with daily feed consumption precisely recorded for each tank. Freshwater was prepared by aerating tap water for 24 h, while saline-alkaline water was prepared by mixing NaHCO_3_, tap water, and seawater, followed by aeration for 24 h. The 70% of water volume was replaced daily, maintaining the water quality parameters at 28.0–31.0 °C and dissolved oxygen levels above 7.0 mg/L.

### Sample collection and calculations

At the end of the trial, all tilapia were fasted for 24 h and then anesthetized using MS-222 (20–80 mg/L) in each tank [34]. The length, weight, and number of fish were recorded to calculate the survival rate (SR), weight gain rate (WG), and feed conversion ratio (FCR). Tail vein blood was collected from three randomly selected fish per tank via syringes containing heparin sodium. Blood samples were kept at 4 °C overnight and centrifuged at 2,500 r/min and 4 °C for 10 min. The plasma supernatant was stored at −80 °C for further analysis. The liver, gills, intestine and muscles were subsequently collected and immediately stored at −80°C. The liver was weighed to calculate the hepatosomatic index (HSI) and condition factor (CF). The entire sampling process was carried out on ice. Three fish per tank were randomly selected and kept at −20 °C for whole fish body composition.

The SR, WG, FCR, HSI and CF were calculated using the following equations:

$$\mathrm{SR}\;(\%)=100\times\frac{\mathrm{final}\;\mathrm{fish}\;\mathrm{number}}{\mathrm{initial}\;\mathrm{fish}\;\mathrm{number}};$$

$$\mathrm{WG}\;(\%)=100\times\frac{\mathrm{final}\;\mathrm{body}\;\mathrm{weight}-\mathrm{initial}\;\mathrm{body}\;\mathrm{weight}}{\mathrm{initial}\;\mathrm{body}\;\mathrm{weight}};$$

$$\mathrm{FCR}\;(\%)=100\times\frac{\mathrm{feed}\;\mathrm{consumption}}{\mathrm{final}\;\mathrm{biomass}-\mathrm{initial}\;\mathrm{biomass}+\mathrm{dead}\;\mathrm{fish}\;\mathrm{weight}};$$

$$\mathrm{HSI}\;(\%)=100\times\frac{\mathrm{wet}\;\mathrm{hepatopancreas}\;\mathrm{weight}}{\mathrm{wet}\;\mathrm{body}\;\mathrm{weight}\;};$$

$$\mathrm{CF}\;(\mathrm g/\mathrm{cm}^3)=100\times\frac{\mathrm{wet}\;\mathrm{body}\;\mathrm{weight}}{\mathrm{body}\;\mathrm{length}^3}.$$

### Whole-body composition detection

The proximate body chemical compositions and the diets were analyzed according to standard methods [35]. The moisture content was determined in an oven at 105 °C until a constant weight was reached. The crude protein content was measured by the Kjeldahl method using Kjeltec™ 8200 (Foss, Sweden). The crude lipid contents were determined by Soxhlet extraction in ether. The muffle furnace (Thermolyne Corporation, Dubuque, Iowa, USA) was burned at 550 °C for 6 h to measure the total ash content.

### Histological analysis

The gills on the same side and the livers of three fish in each tank were randomly chosen and immersed in 4% paraformaldehyde solution for 48 h. The gills and liver were dehydrated in formaldehyde, washed with chloroform, and embedded into a solid wax block. The wax block was cut into 5 μm thick sections using a microtome and stained with hematoxylin and eosin. Finally, the sections were viewed under an optical microscope (Eclipse 200, Nikon, Japan).

### Biochemical analysis

The activities of catalase (CAT), superoxide dismutase (SOD) and glutathione peroxidase (GSH-PX) and the content of malonaldehyde (MDA) in the liver were detected with kits purchased from Nanjing Jiancheng Bioengineering Institute (Nanjing, China). The kits used for the determination of plasma glucose, liver glycogen and muscle glycogen contents were also purchased from Nanjing Jiancheng Bioengineering Institute. The specific operating steps were carried out according to the manufacturer’s instructions.

### Quantitative real time PCR

According to the manufacturer’s protocol, total RNA from the livers was extracted by TRIzol reagent (GLPBIO, USA). After the quantity and quality control of the total RNA, reverse transcription into cDNA was performed using a reverse transcription kit (Biosharp, China). All operations were carried out according to the manufacturer's procedures. The primers designed by Primer 5 software for quantitative real-time polymerase chain reaction (qRT-PCR) are shown in Table 2, and *actb* (actin beta) was used as the internal reference gene. Gene expression was detected by qRT-PCR. The relative expression (fold changes) of the target gene was estimated by using the 2^−ΔΔCt^ method [36].

**Table 2:** List of the *O. niloticus* primers used for qRT-PCR

Gene	Forward primer (5'→3')	Reverse primer (5'→3')	Product size, bp	GenBank
*actb*	AGCCTTCCTTCCTTGGTATGGAAT	TGTTGGCGTACAGGTCCTTACG	102	KJ126772.1
*gs*	CCTCACTCTGCGCTGTTATTC	CAGCGGCATGCCTTCAGTTT	100	XM_013276796.3
*glut 2*	CATTGGCATTCTAATCAGCCAGGT	TTGTAATATTGCTGGCGCTCCA	106	XM_003442884.5
*hk*	TTCCTCTGGGCTTCACCTTCT	ATCTTCCCCTTCGCAGTCTGT	109	XM_039611531.1
*pk*	CAGCATAATCTGCACCATCGGT	ATGAGAGAAGTTAAGACGGGCGA	100	XM_005472621.3
*cs*	CGACTGGTCCCACAACTTCA	GAGCACTAACATTGCCTCCTT	116	XM_003438897
*idh*	ACGCATCGCTGAGTACGCCTT	AGACCGTCTGACATCCGCATGA	99	XM_003437590.5
*pepck*	TGGAAGAACAAACCTTGGCG	TGGGTCAATAATGGGACACTGTCT	99	XM_003448375
*g6pase*	AGACCTTATTGGTGGGTTCACGA	CTGAAGGACTTCCTGGTCCAGTTT	106	XM_003448671.4

*actb* Actin beta, *gs* Glycogen synthase, *glut 2* Glucose transporter 2, *hk* Hexokinase, *pk* Pyruvate kinase, *cs* Citrate synthase, *idh* Isocitrate dehydrogenase, *pepck* Phosphoenolpyruvate carboxykinase, *g6pase* Glucose-6-phosphatase

### Intestinal flora sequencing and analysis

According to the difference in growth performance of tilapia after 50 d of culture, intestinal samples of 5 fish in each group were randomly selected for 16S rDNA sequencing (FW-LP, FW-HP, SW-LP and SW-HP). The sequencing work was completed by Novo Magic Technology Co., Ltd. (Beijing, China). The raw sequences were clustered into operational taxonomic units (OTUs) using the Uparse algorithm (Uparse v7.0.1001, http://www.drive5.com/uparse/) and subsequently annotated. Taxonomic annotation was conducted using the Mothur method with reference to the SSUrRNA database from SILVA version 138.1 (http://www.arb-silva.de/).

### Liver transcriptome analysis

Total RNA was extracted from liver tissues using TRIzol^®^ Reagent (Invitrogen, USA) following the manufacturer’s instructions. The quantity, quality and integrity of the RNA were assessed using the RNA Nano 6000 Assay Kit of the Bioanalyzer 5400 system (Agilent Technologies, CA, USA). One microgram of total RNA was used to construct a sequencing library using the NEBNext^®^ Ultra™ RNA Library Prep Kit for Illumina^®^ (New England Biolabs, USA). After quantification by a Qubit 2.0 fluorometer, the paired-end libraries were sequenced on an Illumina NovaSeq 6000 platform (Tianjin Novogene Bioinformatics Technology Co., Ltd., China). The raw sequence data were quality-controlled using Fastp software (version 0.19.7). Clean data (clean reads) were obtained by removing adaptor sequences, poly-N sequences, and low-quality sequences from the raw data. Moreover, the Q20, Q30, and GC contents of the clean reads were calculated. The index of the reference genome was built, and all paired-end clean reads were separately aligned to the *S. chuatsi* genome using HISAT2 software (v2.0.5). The mapped reads of each sample were assembled by StringTie (v1.3.3b) following a reference-based approach. The abundance of each transcript was calculated according to the fragments per kilobase of exon model per million mapped fragments (FPKM) method. Genes with |log_2_(fold change)|> 1 and adjusted *P* values < 0.05 between libraries were considered differentially expressed genes (DEGs). The DEG analysis was performed using the DESeq2 R package. Subsequently, the Gene Ontology (GO) functional terms and KEGG pathway enrichment of the DEGs were subsequently performed by the clusterProfiler R package (3.8.1), and a corrected *P* value < 0.05 was considered statistically significant. Transcriptome data analysis using the online platform provided by Novo Magic Technology Co., Ltd. (https://magic.novogene.com).

### Statistical analysis

All statistical analysis were performed using SPSS Statistics 19.0 software. All the data met the normal distribution and variance homogeneity test. Two-factor analysis of variance was used to analyze the main effects and interactions of the salinity-alkalinity stress and protein-to-carbohydrate ratios. One-way analysis of variance followed by Duncan's multiple comparison test was used to determine all data. *P* < 0.05 indicated that the difference was statistically significant. All data were presented as the mean ± standard error of the mean (SEM). Correlation network heatmap analysis was performed using online software (https://www.omicshare.com).

## Results

### Growth performance

The growth performance of fish in each group is presented in Table 3. No significant difference was found in SR among all groups (*P* > 0.05). The WG, FCR and HSI were significantly influenced by salinity-alkalinity, N:C ratios and their interaction (*P* < 0.05). The CF was markedly affected by salinity-alkalinity (*P* < 0.05). For the same diets, tilapia in freshwater had significantly greater WG and lower FCR than those in saline-alkaline water (*P* < 0.05). Dramatically greater CF was found in tilapia fed the HP diet in freshwater than in those fed the saline-alkaline water (*P* < 0.05). However, tilapia in saline-alkaline water had markedly higher HSI than those in freshwater except for tilapia fed the HP diet (*P* < 0.05). Under freshwater conditions, the highest WG was observed in tilapia fed the MP diet (*P* < 0.05), and tilapia fed the LP diet had the highest FCR and HSI (*P* < 0.05). Interestingly, under salinity-alkalinity stress, tilapia fed the LP diet had the highest WG, HSI, CF and lowest FCR compared with tilapia fed the other two diets (*P* < 0.05).

**Table 3:** Growth performance of *O. niloticus* fed different experimental diets

Groups	SR, %	WG, %	FCR, %	HSI, %	CF, g/cm^3^
FW–LP	93.33 ± 2.88	6,075.29 ± 210.89^B,*^	123.16 ± 2.60^A^	1.80 ± 0.03^A^	3.27 ± 0.04
FW–MP	89.33 ± 2.18	7,554.11 ± 100.25^A,*^	108.25 ± 2.27^B^	1.56 ± 0.05^B^	3.24 ± 0.04
FW–HP	90.67 ± 2.88	5,842.38 ± 106.21^B,*^	121.68 ± 4.76^AB^	1.71 ± 0.04^AB^	3.40 ± 0.07^*^
SW–LP	94.67 ± 1.09	3,120.57 ± 103.37^a^	178.42 ± 5.58^c,*^	2.08 ± 0.05^a,*^	3.24 ± 0.02^a^
SW–MP	94.67 ± 1.09	2,115.88 ± 19.72^b^	233.39 ± 5.51^b,*^	2.25 ± 0.06^a,*^	3.21 ± 0.03^ab^
SW–HP	93.33 ± 1.09	1,427.59 ± 42.24^c^	259.74 ± 6.72^a,*^	1.83 ± 0.01^b^	3.13 ± 0.01^b^
Two-way ANOVA (*P *value)					
Salinity-alkalinity	0.153	< 0.001	< 0.001	< 0.001	0.018
Protein/Carbohydrate	0.661	< 0.001	< 0.001	0.017	0.772
Interaction	0.723	< 0.001	< 0.001	< 0.001	0.061

*FW* Freshwater, *SW* Saline-alkaline water, *LP* 27% protein with 35% carbohydrate, *MP* 35% protein with 25% carbohydrate, *HP* 42% protein with 15% carbohydrate

*SR* Survival rate, *WG* Weight gain rate, *FCR* Feed conversion ratio, *HSI* Hepatosomatic index, *CF* Condition factor

Data are expressed as the mean ± SEM (standard error of the mean) (*n* = 3, replicate tanks)

^A,B^Indicates a significant difference between dietary protein to carbohydrate ratios within the fresh water (*P* < 0.05)

^a–c^Indicates a significant difference between dietary protein to carbohydrate ratios within the saline-alkaline water (*P* < 0.05)

^∗^Indicates a significant difference between alkalinity levels within the same protein/carbohydrate ratios (*P* < 0.05)

### Whole-body proximate composition

The effects of different experimental groups on the whole-body proximate composition are shown in Table 4. The moisture content was greatly influenced by the N:C ratios (*P* < 0.05). The crude protein content was markedly affected by the salinity-alkalinity and N:C ratios (*P* < 0.05). The crude lipid content was significantly affected by the salinity-alkalinity, N:C ratios and their interaction (*P* < 0.05). The ash content was markedly influenced by the salinity-alkalinity and the interaction between salinity-alkalinity and N:C ratios (*P* < 0.05). For the same diets, tilapia in freshwater had significantly higher crude lipid content contents than those in saline-alkaline water except for tilapia fed the LP diet (*P* < 0.05), while tilapia fed the MP diet in saline-alkaline water had markedly higher ash contents than those in freshwater (*P* < 0.05). Under freshwater conditions, tilapia fed the LP diet had the lowest moisture content and the highest ash content (*P* < 0.05). In addition, under salinity-alkalinity stress, the lowest moisture content and the highest crude protein and lipid contents were found in tilapia fed the LP diet (*P* < 0.05).

**Table 4:** Proximate composition of *O. niloticus* fed different experimental diets with different salinity-alkalinity ratios

Diets	Moisture, %	Crude protein, %	Crude lipid, %	Ash, %
FW–LP	72.94 ± 0.40^B^	18.20 ± 0.19	8.52 ± 0.45	3.20 ± 0.03^A^
FW–MP	73.80 ± 0.21^AB^	17.30 ± 0.38	8.35 ± 0.28^*^	3.01 ± 0.02^AB^
FW–HP	74.94 ± 0.15^A^	17.36 ± 0.26	8.09 ± 0.57^*^	2.89 ± 0.10^B^
SW–LP	72.39 ± 0.14^b^	17.68 ± 0.30^a^	9.23 ± 0.12^a^	3.11 ± 0.07
SW–MP	74.80 ± 0.45^a^	16.49 ± 0.12^ab^	6.67 ± 0.35^b^	3.35 ± 0.01^*^
SW–HP	75.70 ± 0.38^a^	16.06 ± 0.46^b^	5.78 ± 0.15^b^	3.31 ± 0.10
Two-way ANOVA (*P* value)				
Salinity-alkalinity	0.225	0.014	0.010	0.005
Protein/Carbohydrate	< 0.001	0.014	0.002	0.651
Interaction	0.139	0.585	0.011	0.019

*FW* Freshwater, *SW* Saline-alkaline water, *LP* 27% protein with 35% carbohydrate, *MP* 35% protein with 25% carbohydrate, *HP* 42% protein with 15% carbohydrate

Data are expressed as the mean ± SEM (standard error of the mean) (*n* = 3, replicate tanks)

^A,B^Indicates a significant difference between dietary protein to carbohydrate ratios within the fresh water (*P* < 0.05)

^a,b^Indicates a significant difference between dietary protein to carbohydrate ratios within the saline-alkaline water (*P* < 0.05)

^∗^Indicates a significant difference between alkalinity levels within the same protein-carbohydrate ratios (*P* < 0.05)

### Gill and liver histology

**Fig. 1:**
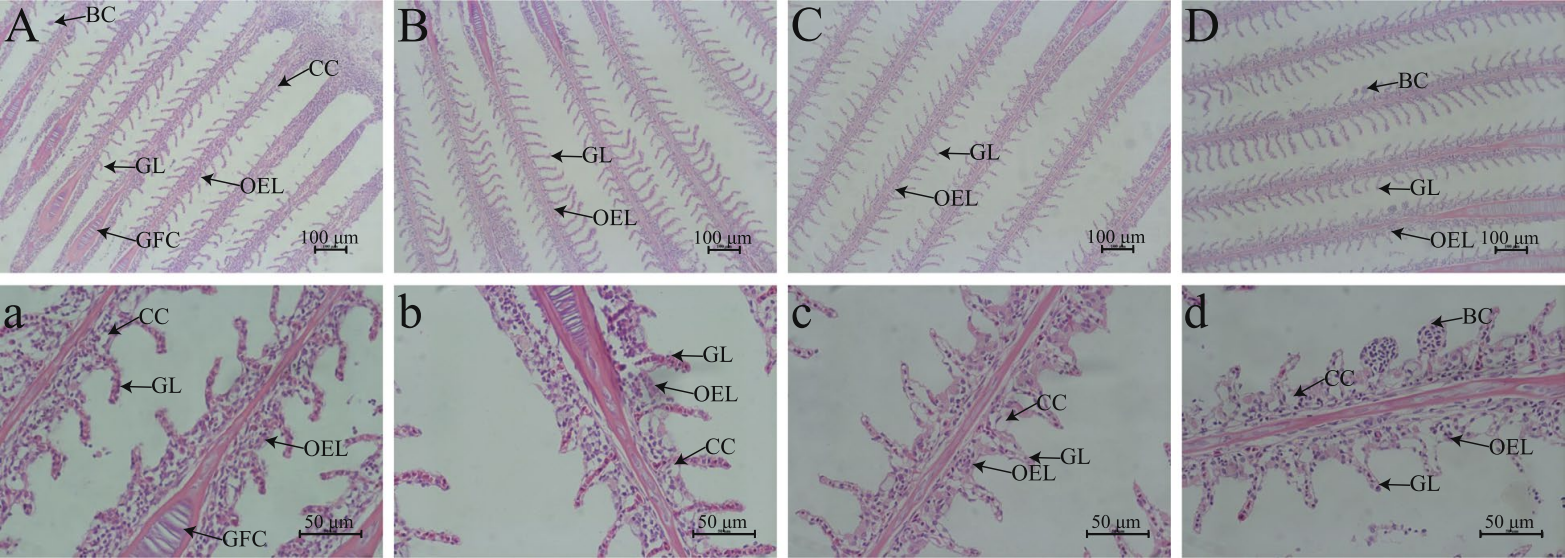
The effects of *O. niloticus* fed different diets on gill structure parameters in freshwater or saline-alkaline water for 50 d. **A** and **a** Staining section of gill structure of tilapia fed the LP diet in freshwater. **B** and **b** Staining section of gill structure of tilapia fed the HP diet in freshwater. **C** and **c** Staining section of gill structure of tilapia fed the LP diet in saline-alkaline water. **D** and **d** Staining section of gill structure of tilapia fed the HP diet in saline-alkaline water. **A–D**, scale bar = 100 μm; **a–d**, scale bar = 50 μm. *BC* Blood cell, *GL* Gill lamella, *GFC* Gill filament cartilage,* OEL* outer epithelial layer, *CC* Chloride cell

**Fig. 2:**
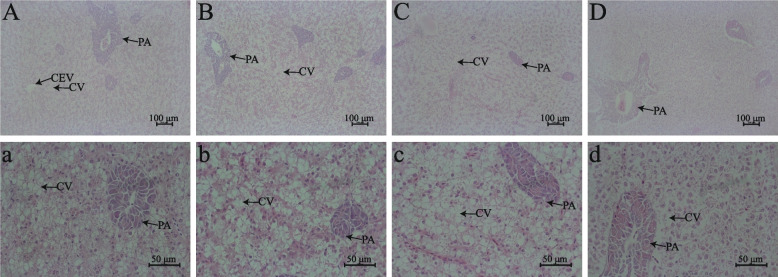
The effects of *O. niloticus* fed different diets on liver structure parameters in fresh water or saline-alkaline water for 50 d. **A** and **a** Staining section of liver structure of tilapia fed the LP diet in freshwater. **B** and **b** Staining section of liver structure of tilapia fed the HP diet in freshwater. **C** and **c** Staining section of liver structure of tilapia fed the LP diet in saline-alkaline water. **D** and **d** Staining section of liver structure of tilapia fed the HP diet in saline-alkaline water. **A–D**, scale bar = 100 μm; **a**–**d**, scale bar = 50 μm. *CEV* Central veins, *CV* Cytoplasmic vacuolation, *PA* Pancreatic acini

Gills are important organs for fish to regulate respiration and maintain osmotic balance. Compared with the freshwater groups (Fig. 1A, B, a and b), tilapia in saline-alkaline water had shorter gill lamellae and more chlorine cells on the gill (Fig. 1C, D, c and d). In addition, the distribution of red blood cells in the gills of tilapia in saline-alkaline water was disordered. Under salinity-alkalinity stress, partial erythrocyte accumulation was observed in tilapia fed the HP diet. The liver is an important organ of fish metabolism. Compared with tilapia fed the HP diet (Fig. 2B, D, b and d), cytoplasmic vacuolation was observed in tilapia fed the LP diet (Fig. 2A, C, a and c). In addition, for the same diets, more severe cytoplasmic vacuolation was observed in tilapia in saline-alkaline water.

### Antioxidant capacity

**Fig. 3:**
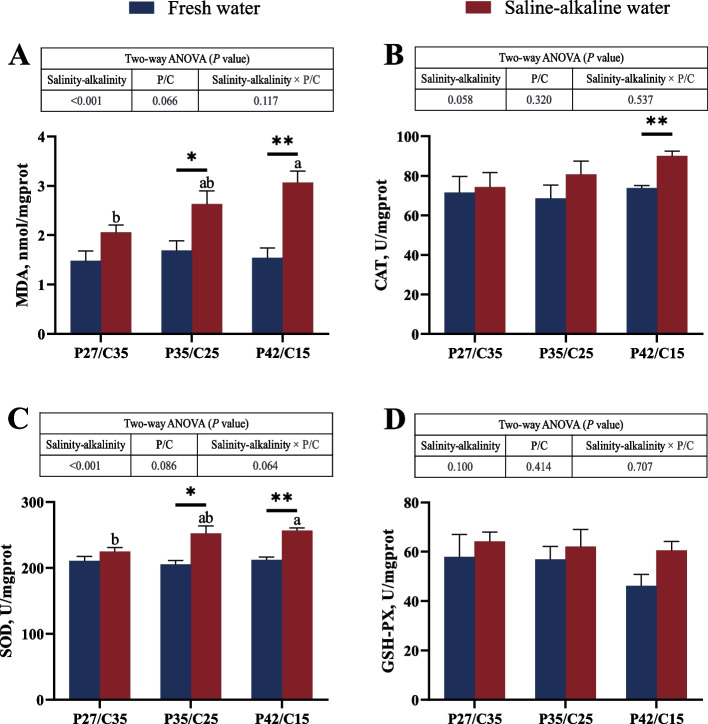
Activities of MDA (**A**), CAT (**B**), SOD (**C**) and GSH-PX (**D**) in the liver of *O. niloticus* fed different dietary protein to carbohydrate ratios in fresh water or saline-alkaline water for 50 d. All data are presented as the mean ± SEM (*n* = 3). ^a,b^Different letters indicate significant differences among the saline-alkaline water groups (*P* < 0.05). ^*^Indicates a significant difference between different salinity-alkalinity levels within the same diet protein-carbohydrate ratios (*P* < 0.05). ^**^Indicates a significant difference between different salinity-alkalinity levels within the same diet protein-carbohydrate ratios (*P* < 0.01)

There were significant main effects of salinity-alkalinity on the MDA content and SOD activity (*P* < 0.05). Tilapia in saline-alkaline water had a significantly higher MDA content and SOD activity than those in freshwater, except for tilapia fed the LP diet (*P* < 0.05, Fig. 3A and C). In addition, tilapia fed the HP diet in saline-alkaline water had markedly higher CAT activity than those in freshwater (*P* < 0.05, Fig. 3B). No marked difference was shown in the GSH-PX activity (*P* > 0.05, Fig. 3D). Notably, under salinity-alkalinity stress, tilapia fed the HP diet had the highest MDA content and SOD activity (*P* > 0.05).

### Plasma glucose and tissue glycogen

**Fig. 4:**
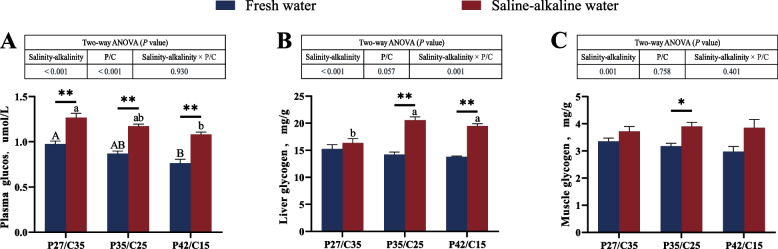
Plasma glucose (**A**), liver glycogen (**B**) and muscle glycogen (**C**) contents of *O. niloticus* fed different dietary protein to carbohydrate ratios in fresh water or saline-alkaline water for 50 d. All data are presented as the mean ± SEM (*n* = 3). ^A,B^Different letters indicate significant differences among the fresh water groups (*P* < 0.05). ^a,b^Different letters indicate significant differences among the saline-alkaline water groups (*P* < 0.05). ^*^Indicates a significant difference between different salinity-alkalinity levels within the same diet protein-carbohydrate ratios (*P* < 0.05). ^**^Indicates a significant difference between different salinity-alkalinity levels within the same diet protein-carbohydrate ratios (*P* < 0.01)

A significant main effect of the salinity-alkalinity and N:C ratios was found on the plasma glycose (*P* < 0.05). Liver glycogen was markedly influenced by salinity-alkalinity and the interaction between salinity-alkalinity and N:C ratios (*P* < 0.05), and muscle glycogen was significantly affected by salinity-alkalinity (*P* < 0.05). Regardless of diet, tilapia in saline-alkaline water had significantly higher plasma glycose levels (*P* < 0.05, Fig. 4A). Compared with tilapia in freshwater, tilapia in saline-alkaline water had markedly higher liver glycose levels, except for tilapia fed the LP diet (*P* < 0.05, Fig. 4B). In addition, markedly higher muscle glycogen was observed in tilapia fed the MP diet in saline-alkaline water than those in freshwater (*P* < 0.05, Fig. 4C). In freshwater, tilapia fed the LP diet had the highest plasma glucose (*P* < 0.05). Tilapia fed the LP diet had the highest plasma glucose level and the lowest liver glycogen level under salinity-alkalinity stress (*P* < 0.05). In the same water environment, the three diets had no significant effects on the muscle glycogen level of tilapia (*P* > 0.05).

### Expression of glucose metabolism-related genes in the liver

**Fig. 5:**
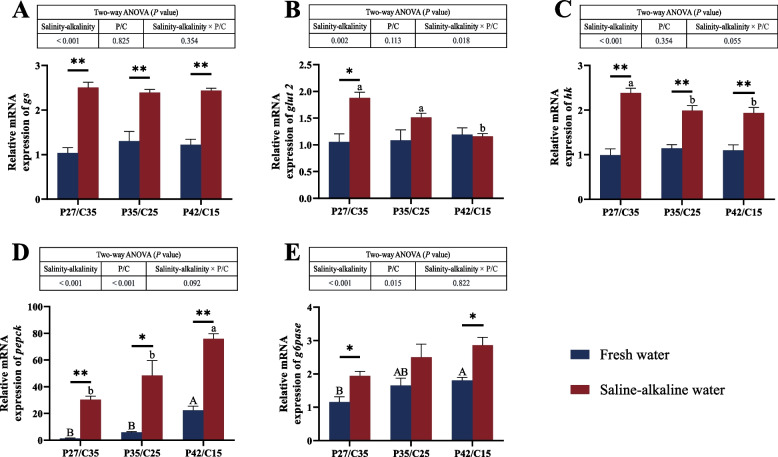
Expression of genes related to glycometabolism [glycogen synthase (**A**), glucose transporter 2 (**B**), hexokinase (**C**), pyruvate kinase (**D**), citrate synthase (**E**), isocitrate dehydrogenase (**F**), phosphoenolpyruvate carboxykinase (**G**), glucose-6-phosphatase (**H**)] in the liver of *O. niloticus* fed different diets in freshwater or saline-alkaline water for 50 d. All data are presented as the mean ± SEM (*n* = 3). ^A,B^Different letters indicate significant differences among the fresh water groups (*P* < 0.05). ^a,b^Different letters indicate significant differences among the saline-alkaline water groups (*P* < 0.05). ^*^Indicates a significant difference between different salinity-alkalinity levels within the same diet protein-carbohydrate ratios (*P* < 0.05). ^**^Indicates a significant difference between different salinity-alkalinity levels within the same diet protein-carbohydrate ratios (*P* < 0.01). *gs*, glycogen synthase; *glut 2*, glucose transporter 2; *hk*, hexokinase; *pk*, pyruvate kinase; *cs*, citrate synthase; *idh*, isocitrate dehydrogenase; *pepck*, phosphoenolpyruvate carboxykinase; *g6pase*, glucose-6-phosphatase

The expression of genes related to glycogen synthase (*gs*), hexokinase (*hk*), pyruvate kinase (*pk*), citrate synthase (*cs*) and isocitrate dehydrogenase (*idh*) was significantly influenced by salinity-alkalinity (*P* < 0.05). The mRNA level of glucose transporter 2 (*glut 2*) was markedly affected by salinity-alkalinity and the interaction between salinity-alkalinity and the N:C ratios (*P* < 0.05), and the mRNA levels of phosphoenolpyruvate carboxykinase (*pepck*) and glucose-6-phosphatase (*g6pase*) were markedly influenced by salinity-alkalinity and the N:C ratios (*P* < 0.05). For the same diets, tilapia in saline-alkaline water had markedly higher expression levels of *gs*, *hk* and *pepck* than those in freshwater (*P* < 0.05, Fig. 5A, C and G). The expression level of *glut 2* was significantly upregulated in tilapia fed the LP diet in saline-alkaline water compared with those in freshwater (*P* < 0.05, Fig. 5B), and the expression level of *idh* was significantly upregulated in tilapia fed the MP diet in saline-alkaline water compared with those in freshwater (*P* < 0.05, Fig. 5F). No marked difference was found in the mRNA level of *pk* (*P* < 0.05, Fig. 5D). In addition, tilapia in saline-alkaline water had a significantly higher *cs* expression level than those in freshwater except for tilapia fed the HP diet (*P* < 0.05, Fig. 5E), and tilapia in saline-alkaline water had a markedly higher *g6pase* expression level than those in freshwater except for tilapia fed the MP diet (*P* < 0.05, Fig. 5H). In fresh water, the highest expression levels of *pepck* and *g6pase* were observed in tilapia fed the HP diet (*P* < 0.05). In addition, tilapia fed the LP diet had the highest mRNA levels of *glut 2* and *hk* and the lowest mRNA levels of *cs* and *pepck* under salinity-alkalinity stress (*P* < 0.05).

### Associations among approximate composition, antioxidant activity, glycogen, metabolism and growth

**Fig. 6:**
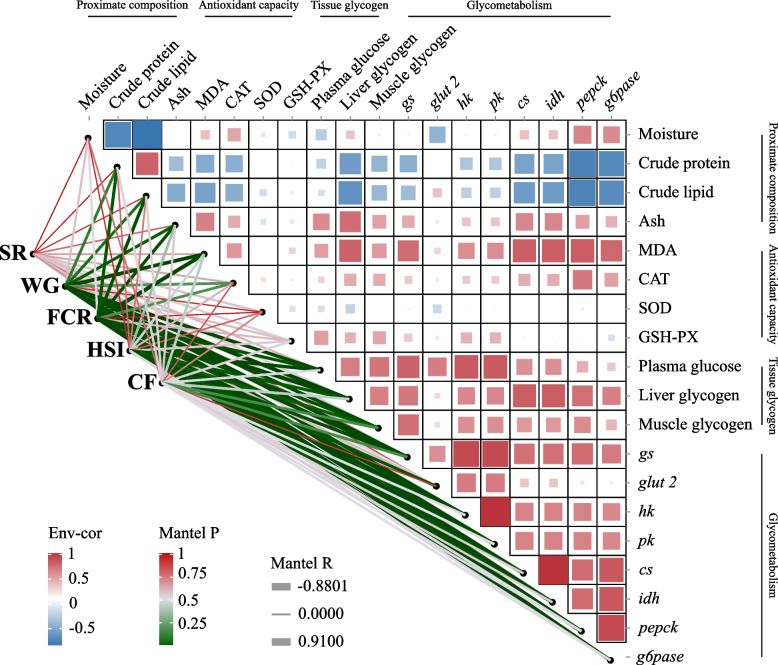
Correlations among growth performance, biochemical analysis, and qRT-PCR. The edge width of lines refers to Mantel’s r for the statistics of corresponding distance correlations, and the color of lines represents the statistical significance

The Fig. 6 shows that the growth performance of tilapia was significantly associated with proximate composition, antioxidant capacity, tissue glycogen content, and glycometabolism. The SR was not significantly correlated with the proximate whole fish composition, antioxidant capacity, tissue glycogen level, or glycometabolism (*P* > 0.05). The WG was significantly negatively correlated with the MDA content and the expression levels of genes related to glucose metabolism (*P* < 0.05). The FCR was significantly positively correlated with whole-fish crude protein, crude fat, and MDA contents, as well as with the expression levels of genes related to glucose metabolism (*P* < 0.05). The HSI was significantly positively correlated with the tissue glycogen content and the expression levels of genes involved in the glycolytic pathway (*P* < 0.05).

### Gut microbiota analysis

An average of 1,929,544 tags per sample were detected, and an average of 1,824,109 valid data points were obtained through quality control. After the sequences were clustered into OTUs, with 95% consistency (identity), a total of 3,647 OTUs were obtained.

#### Gut microbiota diversity

**Fig. 7:**
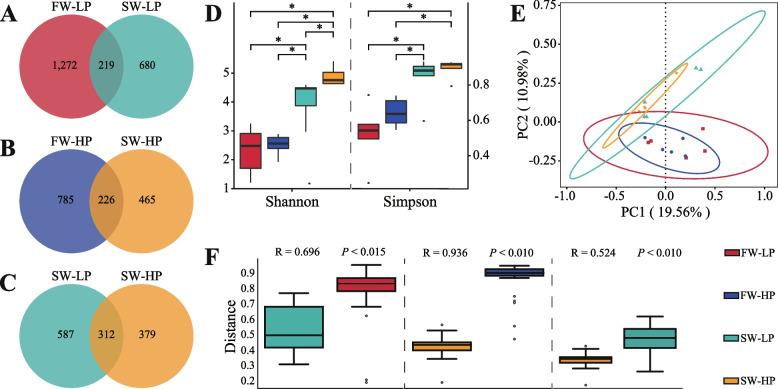
Changes in the gut microbiota diversity of *O. niloticus* fed different diets in freshwater or saline-alkaline water for 50 d. **A**–**C** Venn diagram showing the numbers of shared and unique ASVs. **D** The Shannon and Simpson indices of α-diversity. **E** PCoA score plot. **F** Analysis of similarity (ANOSIM) of gut microbiota. All data are presented as the mean ± SEM (*n* = 5). Statistical analysis was performed using one-way analysis of variance, and statistical significance is indicated by * (*P* < 0.05)

The FW-LP and SW-LP groups (Fig. 7A), the FW-HP and SW-HP groups (Fig. 7B) and the SW-LP and SW-HP groups (Fig. 7C) shared 219, 226 and 312 OTUs, respectively. According to the α-diversity results, there were significant differences in the diversity and abundance of the microbiota in all groups (Fig. 7D). The β-diversity results showed that the microbiota of the fresh water groups and saline-alkaline water groups were obviously separated (Fig. 7E). In addition, based on the results of the similarity analysis (ANOSIM), the intestinal microbiota structure of fish in different experimental groups changed significantly (R > 0, *P* < 0.05, Fig. 7F).

**Fig. 8:**
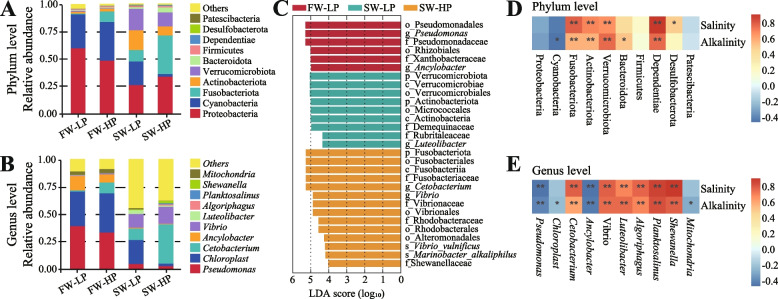
Changes in the gut microbiota composition and characteristics of *O. niloticus* fed different diets in freshwater or saline-alkaline water for 50 d. **A** Microbiota composition at the phylum level with relative abundances in the top ten. **B** Microbiota composition at the genus level with relative abundances in the top ten. **C** Bacterial taxa differentially expressed in each group identified by LEfSe using an LDA score threshold of > 4. **D** Correlation analysis of environmental factors at the phylum level. **E** Correlation analysis of environmental factors at the genus level. All data are presented as the mean ± SEM (*n* = 5). ^*^P < 0.05, ^**^P < 0.01

At the phylum level, Proteobacteria was the most abundant phylum in all samples (Fig. 8A). At the genus level, compared with those in the freshwater groups, the relative abundances of *Pseudomonas* and *Chloroplast* were obviously decreased, and the relative abundances of *Cetobacterium* and *Vibrio* were obviously increased (Fig. 8B). LEfSe analysis revealed that some bacterial taxa at different classification levels were significantly enriched in each group (Fig. 8C). The results of the correlation analysis of environmental factors at the phylum level showed that the relative abundances of Verrucomicrobiota, Actinobacteriota and Fusobacteriota markedly increased under salinity-alkalinity stress (*P* < 0.05, Fig. 8D). The results of the correlation analysis of environmental factors at the genus level showed that the relative abundances of *Shewanella*, *Planktosalinus*, *Vibrio* and other genera markedly increased under salinity-alkalinity stress (*P* < 0.05, Fig. 8E).

#### Gut microbiota functional prediction

**Fig. 9:**
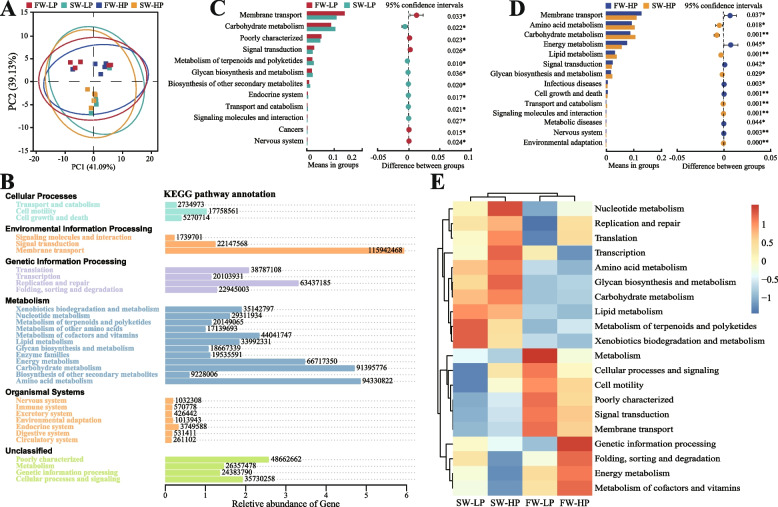
Effects of *O. niloticus* fed different diets in freshwater or saline-alkaline water on gut microbiota function. **A** PCA score plot. **B** The statistical map of gene prediction results reveals the annotated proportion of genes. **C** and **D** The abundance ratio of gut microbiota and level 2 functional prediction among groups. ^*^P < 0.05, ^**^P < 0.01. **E** KEGG level 2 functional relative abundance clustering heatmap. All data are presented as the mean ± SEM (*n* = 5)

The results of the PCA score plot for functional prediction showed that freshwater groups presented similar functional OTUs, while saline-alkali groups had similar functional OTUs (Fig. 9A). The KEGG pathways enriched in all groups were classified into six categories at KEGG level 1: Cellular Processing, Environmental Information Processing, Genetic Information Processing, Metabolism, Organismal Systems and unclassified (Fig. 9B). At KEGG level 2, compared with the FW-LP group, the SW-LP group had significant differences in terms of membrane transport, carbohydrate metabolism, and poor characterization (Fig. 9C), and the SW-HP group had marked differences in terms of membrane transport, amino acid metabolism, and carbohydrate metabolism compared with the FW-HP group (Fig. 9D). The KEGG heatmap of functional prediction showed that nucleotide metabolism, replication and repair, translation and other processes were significantly enriched in the saline-alkali groups (Fig. 9E).

### Transcriptome analysis

RNA-seq analysis of the livers of 12 fishes from the FW-LP, FW-HP, SW-LP and SW-HP groups yielded 541,114,934 total raw reads, yielding 534,288,954 total clean reads after optimization and quality control. The Q30% was above 93.5%. The total mapped ratios of the clean reads with the reference genome of tilapia ranged from 89.04% to 93.50%.

**Fig. 10:**
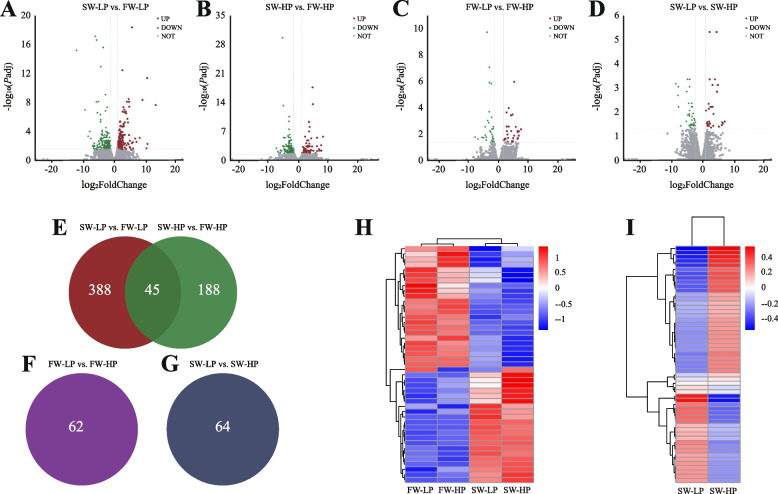
DEG analysis of *O. niloticus* fed different diets in freshwater or saline-alkaline water for 50 d. **A**–**D** Differential gene volcano map of each comparison group. **E**–**G** Venn diagram showing the number of shared and unique DEGs in the comparison group. **H** and **I** Heatmap of DEG cluster analysis. All data are presented as the mean ± SEM (*n* = 3)

Among all expressed genes, the SW-LP group contained 226 upregulated and 207 downregulated genes compared with the FW-LP group (Fig. 10A). Compared with the FW-HP group, the SW-HP group contained 93 upregulated and 140 downregulated genes (Fig. 10B). The FW-LP group contained 30 upregulated and 32 downregulated genes compared with the FW-HP group (Fig. 10C). The SW-LP group contained 29 upregulated and 35 downregulated genes compared with the SW-HP group (Fig. 10D). Specifically, compared with the freshwater groups, 45 shared DEGs were found in the saline-alkaline water (Fig. 10E). In addition, compared with the FW-HP group, 62 DEGs were found in the FW-LP group (Fig. 10F), and 64 DEGs were found in the SW-LP group compared with the SW-HP group (Fig. 10G). For the purpose of the experiment, 45 shared DEGs and 64 DEGs were cluster analyzed to obtain the corresponding DEG cluster heatmap (Fig. 10H and I). After that, genes in each cluster were subjected to functional enrichment analysis.

#### KEGG functional enrichment analysis of DEGs

**Fig. 11:**
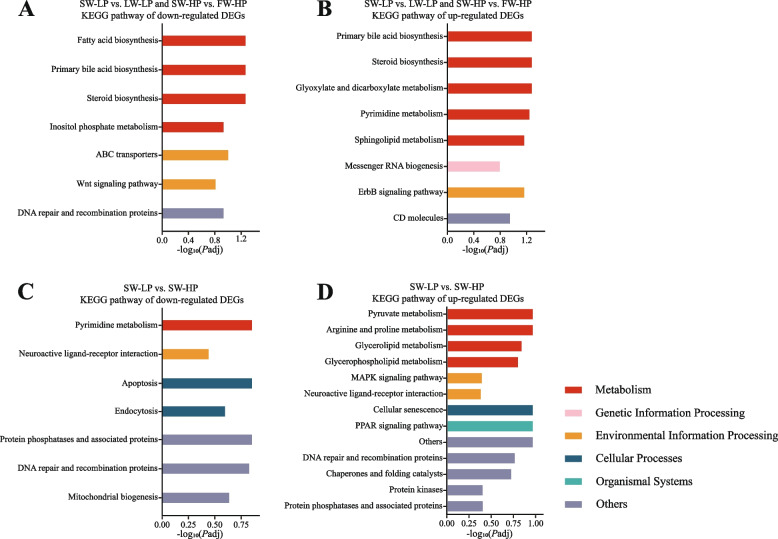
KEGG pathway analysis of *O. niloticus* fed different diets in freshwater or saline-alkaline water for 50 d. **A**–**D** Significantly enriched KEGG pathways in the comparison group. All data are presented as the mean ± SEM (*n* = 3)

Compared with freshwater groups, 45 shared DEGs in saline-alkali groups were clustered on the KEGG pathways “metabolism”, “genetic information processing” and “environmental information processing”. Specifically, the downregulated DEGs were enriched in fatty acid biosynthesis, primary bile acid biosynthesis, and steroid biosynthesis (Fig. 11A), and the upregulated DEGs were enriched in primary bile acid biosynthesis, steroid biosynthesis, and glyoxylate and dicarboxylate metabolism (Fig. 11B). In addition, compared with the SW-HP group, 64 DEGs in the SW-LP group were clustered on the KEGG pathways “metabolism”, “environmental information processing”, “cellular processes” and “organismal systems”. These downregulated DEGs were enriched in pyrimidine metabolism, neuroactive ligand-receptor, apoptosis and endocytosis (Fig. 11C), and these upregulated DEGs were mainly enriched in pyruvate metabolism, arginine and proline metabolism, and glycerolipid metabolism (Fig. 11D).

#### Gene set enrichment analysis (GSEA)

**Fig. 12:**
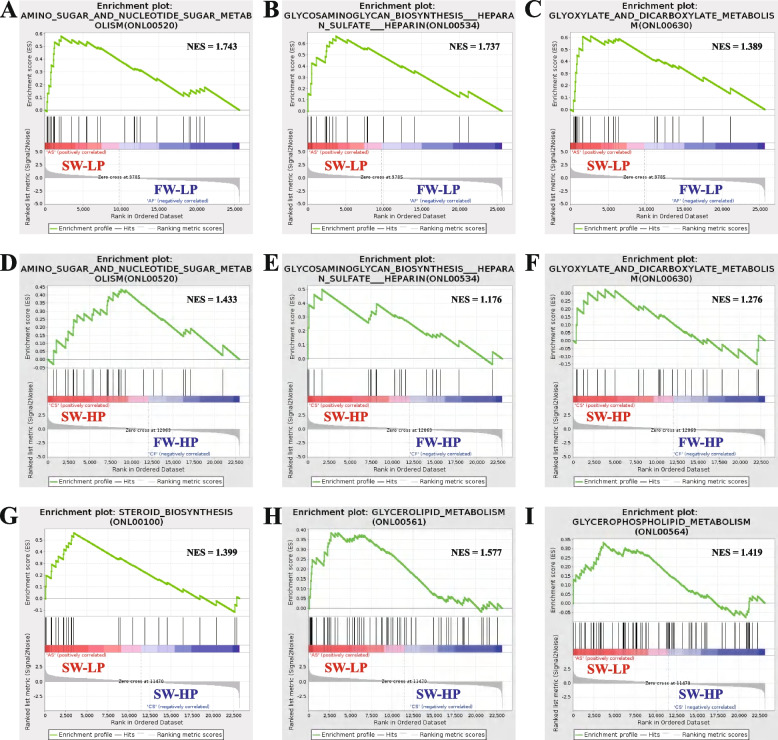
GSEA of *O. niloticus* fed different diets in freshwater or saline-alkaline water for 50 d. **A** SW-LP vs. FW-LP, amino sugar and nucleotide sugar metabolism. **B** SW-LP vs FW-LP, glycosaminoglycan biosynthesis heparan sulfate heparin. **C** SW-LP vs. FW-LP, glyoxylate and dicarboxylate metabolism. **D** SW-HP vs. FW-HP, amino sugar and nucleotide sugar metabolism. **E** SW-HP vs. FW-HP, glycosaminoglycan biosynthesis heparan sulfate heparin. **F** SW-HP vs. FW-HP, glyoxylate and dicarboxylate metabolism. **G** SW-LP vs. SW-HP, steroid biosynthesis. **H** SW-LP vs. SW-HP, glycerolipid metabolism. **I** SW-LP vs. SW-HP, glycerophospholipid metabolism

By detecting the expression changes in the entire gene set, GSEA can be used to comprehensively detect genes whose expression differences are not significant but whose overall level of biological significance is important. The results showed that the gene sets associated with the “amino sugar nucleotide sugar metabolism”, “glycosaminoglycan biosynthesis—heparan sulfate and heparin” and “glyoxylate and dicarboxylate metabolism” pathways were upregulated in the saline-alkali groups compared with the freshwater groups (Fig. 12A–F). Interestingly, the gene sets of the “steroid biosynthesis”, “glycerolipid metabolism” and “glycerophospholipid metabolism” pathways were upregulated in the SW-LP group compared with the SW-HP group (Fig. 12G–I).

## Discussion

Under the salinity-alkalinity stress, the acid–base balance, osmotic balance and normal physiological state of fish are severely impaired, which makes it impossible for most fish in saline-alkaline water to survive [6]. As a saline-alkali tolerant fish, tilapia had no significant difference in survival rate between fresh water and saline-alkaline water in this study. Previous studies have shown that fish must expend a large amount of energy to cope with salinity-alkalinity stress, which reduces the energy supply for normal growth [10, 37]. In this study, tilapia in saline-alkaline water had lower WG and SGR and higher FCR than tilapia in fresh water. In general, higher dietary protein levels could promote better growth performance in fish [38–40]. However, under salinity-alkalinity stress, the WG and SGR of tilapia decreased with increasing dietary protein levels in this study. This is because the increase in dietary protein levels may aggravate the ammonia metabolism burden caused by salinity-alkalinity stress, leading to more severe ammonia poisoning and reduced growth performance in aquatic animals [41, 42]. On the other hand, dietary carbohydrate can directly provide energy for osmotic regulation [23, 43]. The high-carbohydrate diet has been shown to increase the HSI in fish [44, 45]. Therefore, tilapia fed the high-carbohydrate diet presented the highest HSI, CF and whole fish crude lipid contents, regardless of whether freshwater or saline-alkaline water was used. However, the results of this study showed that a high carbohydrate diet can significantly improve the growth performance and the crude protein content of whole fish in saline-alkaline water. The crude protein content of whole fish significantly impacts the processing attributes of fish food, and it is also an important factor influencing consumer purchasing decisions [46, 47]. These findings seemed to indicate that high dietary carbohydrate contents were more beneficial for tilapia in saline-alkaline water than in freshwater. In general, reducing dietary protein by increasing dietary carbohydrate is beneficial for promoting the growth of fish under salinity-alkalinity stress.

Gills are in direct contact with the external environment, so the gills are the primary organ affected by the water environment [48]. Studies have shown that salinity stress can cause deformation of the secondary filament epithelium, hyperemia of primary filament vessels and an increased number of chlorine cells [49]. Alkalinity stress led to a reduced number of cells in the interlamellar cell mass, epithelial vacuolation and lamellar vascular congestion in *Lateolabrax maculatus* [14]. In this study, compared with the freshwater groups, the gills of tilapia in saline-alkaline water showed shorter gill flakes, increased chlorine cell numbers and disordered red blood cell distributions. Moreover, the gills of tilapia fed the high-protein diet showed significant red blood cell aggregation in saline-alkaline water in this study, which may be related to ammonia poisoning [50]. Ammonia poisoning can cause blood vessels in gill tissues, which can cause the gill structure to swell, rupture, and even extravasate [51]. In addition, the liver has the function of detoxification and immunity in bony fish [52, 53]. Previous studies reported that salinity-alkalinity stress could severely damage the hepatopancreatic tissue in *Eriocheir sinensis* [54]. In this study, we found that salinity-alkalinity stress can cause obvious liver cytoplasmic vacuolation accompanied by nuclear migration in fish liver, which also indirectly confirmed that salinity-alkalinity stress can affect the metabolism of aquatic animals. Notably, high-carbohydrate diets can also lead to adverse effects such as hepatocyte vacuolation and disruption of hepatic cords [29]. Our results also found that tilapia fed high-protein diets with low carbohydrate contents presented lower levels of hepatocyte vacuolation compared to those fed a low-protein diets with high carbohydrate contents. In conclusion, tilapia fed a high carbohydrate diet with low protein contents increases liver tissue damage in saline-alkaline water, although it may help maintain gill morphology.

Environmental stress can cause oxidative stress in aquatic animals, which in turn causes the accumulation of reactive oxygen species (ROS) in the body and eventually leads to inhibited growth, increased morbidity, and even death [55–57]. An increase in ROS concentrations can cause lipid peroxidation with the generation of MDA, which is a major factor leading to the loss of cell function and DNA damage [42]. In this study, compared with tilapia in freshwater, the MDA content of tilapia in saline-alkaline water was significantly greater, which meant that salinity-alkalinity stress caused oxidative stress. It has been reported that salinity-alkalinity stress can cause oxidative stress and increase MDA contents in *Eriocheir sinensis* [54]. An increase in both dietary carbohydrate and protein can lead to oxidative stress and an increase in the MDA content [58–60]. However, our results indicated that the MDA content in tilapia fed a high-protein diet in saline-alkaline water was significantly higher than that in those fed a high-carbohydrate diet. Therefore, we speculated that adverse effects such as the ammonia metabolic burden caused by a high-protein diet were more harmful to tilapia than adverse effects such as liver metabolic disorders caused by a high-carbohydrate diet under long-term salinity-alkalinity stress. In this study, tilapia fed a high-protein diet had higher MDA contents than tilapia fed a high-carbohydrate diet under salinity-alkalinity stress. Fish can activate the antioxidant enzyme system to eliminate excess ROS and mitigate oxidative damage under stress [61]. The SOD and CAT are the important defensive enzymes that protect organisms against the ROS produced in these organisms [62]. SOD can convert O_2_^−^ into O_2_ and H_2_O_2_, and H_2_O_2_ is decomposed into H_2_O and O_2_ by CAT [42]. In this study, tilapia fed the high-protein diet in saline-alkaline water had markedly higher CAT and SOD activities than those in freshwater. However, there was no significant difference in the CAT or SOD activity of tilapia fed high-carbohydrate diets between saline-alkaline water and freshwater, suggesting that a high-carbohydrate diet may effectively help tilapia resist oxidative stress [63, 64]. Studies have also shown that Wuchang fish (*Megalobrama amblycephala*) fed high-carbohydrate diets can mitigate oxidative stress damage by activating antioxidant pathways [65]. In summary, salinity-alkalinity stress induces oxidative damage in fish, but a high-carbohydrate diet can effectively alleviate this damage by activating antioxidant pathways under salinity-alkalinity stress.

Energy metabolism not only is closely related to the antioxidant capacity of aquatic animals [66–68], but also plays an important role in the adaptation of aquatic animals to salinity-alkalinity stress [13, 14]. The tricarboxylic acid cycle is a key metabolic pathway linking the metabolism of carbohydrates, lipids and proteins, and its upregulation is typically associated with increased energy demand [69, 70]. In this study, the gene expression levels of *cs* and *idh* involved in the tricarboxylic acid cycle in tilapia in saline-alkaline water were significantly higher than those in freshwater tilapia. Studies have shown that carbohydrates are the most direct energy source for fish to cope with osmotic challenges, among which glycolysis is one of the important energy supply pathways [64, 71]. In this study, the expression levels of the rate-limiting genes *hk* and *pk* involved in glycolysis in tilapia in saline-alkaline water were higher than those in freshwater water. Furthermore, as key genes in glycogen synthesis and gluconeogenesis pathways, the mRNA levels of *gs*, *pepeck* and *g6pase* in tilapia in saline-alkaline water were greater than those in freshwater in this study. Although previous studies suggested that the gluconeogenic pathway was not affected by dietary carbohydrate levels [72], this study revealed that tilapia fed the high-protein diet presented increased gene expression of *pepck* and *g6pase* under salinity-alkalinity stress. This finding suggests that fish may require additional carbohydrate to meet heightened energy demands under such conditions. Stored glycogen can provide substrates for glycolysis and oxidative phosphorylation, ultimately providing energy for the organism [73, 74]. Additionally, the increase in glucose content in the body is often accompanied by increased glucose transport capacity [23, 75]. Our results found that tilapia in saline-alkaline water had a higher tissue glycogen content and gene expression of *glut 2* than those in freshwater. These findings further indicated that tilapia needed to improve glucose metabolism to cope with salinity-alkalinity stress. In conclusion, carbohydrates play a pivotal role as an energy source for tilapia under salinity-alkalinity stress, and a high-carbohydrate diet appears to better support the energy requirements of tilapia in saline-alkaline water.

The intestinal microbiota of animals has various effects on regulating the intestinal metabolism, nutrient absorption, immune system development and disease prevention in the host [76]. Studies have shown that the abundance and diversity of the intestinal microbiota in aquatic animals are closely related to the habitat environment [77, 78]. In this study, salinity-alkalinity stress significantly decreased the abundance and diversity of the intestinal microbiota in tilapia. This may be because the saline-alkali environment inhibits the growth of some bacteria [12, 64]. Furthermore, salinity stress and alkalinity stress have been shown to increase the number of pathogenic bacteria and decrease the number of beneficial bacteria in the intestines of aquatic animals [12, 79]. In this study, salinity-alkalinity stress led to a significant increase in pathogenic bacterial phyla and genera in fish, such as Proteobacteria, *Vibrio*, and *Shewanella*. In particular, regardless of the high-carbohydrate diet or high-protein diet, the Fusobacteriota, Bacteroidota, *Cetobacterium* and mitochondria of tilapia in saline-alkaline water were significantly higher than those in freshwater in this study. Previous studies have reported that the Fusobacteriota, Bacteroidota, *Cetobacterium* and mitochondria play important roles in carbohydrate metabolism in fish [80–83]. Therefore, we speculated that salinity-alkalinity stress promoted the digestion and utilization of carbohydrates by intestinal microorganisms to provide energy for the organism. In the aquaculture industry, the pathogen *Vibrio* can cause infection and inflammation, which can cause economic losses [80]. Notably, our results revealed that the abundance of *Vibrio* in tilapia fed a high-protein diet in saline-alkaline water was significantly higher than those fed a high-carbohydrate diet in saline-alkaline water. These findings suggested that a high-protein diet was more likely to harm the health of tilapia than a high-carbohydrate diet in saline-alkaline water. Generally, an increase in the abundance of *Vibrio* in the intestines leads to an increase in MDA content and oxidative damage in the body, which is consistent with the increase in the MDA content in this study [84]. Overall, salinity-alkalinity stress can increase the abundance of pathogenic bacteria in tilapia. However, high-carbohydrate diets can reduce the abundance of pathogenic bacteria in tilapia, thereby maintaining intestinal health.

In addition to the gut microbiota, the carbohydrate metabolism and lipid metabolism pathways of tilapia in saline-alkaline water were significantly enriched compared with those in freshwater by the functional prediction of the intestinal microbiota. The functional prediction of the intestinal microbiota also indicated that tilapia fed the high-protein diet in saline-alkaline water presented lower energy metabolism levels than those in freshwater in this study, which meant that high-protein diets disrupt the normal energy metabolism of tilapia under salinity-alkalinity stress. These results suggested that fish tend to rely more on nonprotein substances for energy under salinity-alkalinity stress. This occurred because both osmotic regulation and ammonia poisoning caused by salinity-alkalinity stress can increase the demand for energy [85–87]. However, protein catabolism aggravates the ammonia metabolism burden of fish under salinity-alkalinity stress, and excessive ammonia accumulation can cause energy metabolism disorders in aquatic animals [86, 88]. In addition, at the liver transcriptomic level, we also observed that the DEGs among the groups were enriched mainly in metabolism-related pathways in this study. Among them, steroid biosynthesis, glyoxylate and dicarboxylate metabolism, and sphingolipid metabolism related to glucose and lipid metabolism were significantly upregulated under salinity-alkalinity stress. In addition, our results shown that pyruvate metabolism, glycerophospholipid metabolism and MAPK signaling pathways were significantly enriched in tilapia fed a high-carbohydrate diet under salinity-alkalinity stress compared with tilapia fed a high-protein diet. Pyruvate metabolism is a key link in cellular energy metabolism, connecting multiple metabolic pathways, such as glycolysis, the citric acid cycle and the respiratory chain [89], and glycerophospholipid metabolism and the MAPK signaling pathway have also been shown to be closely related to osmotic regulation and alkalinity adaptation [14, 90–92]. These findings further support the conclusion of this study that fish prefer to utilize dietary carbohydrates under salinity-alkalinity stress. An adequate energy supply is essential for the survival and development of aquatic animals under stress [10, 93]. This may be because the direct energy supply from carbohydrates is more beneficial for adjusting the ion imbalance caused by salinity-alkalinity, so the fish fed a high-carbohydrate diet in this study had better growth performance. In addition, most scholars believe that the ability to relieve ammonia poisoning is a key factor in determining the tolerance of aquatic animals to salinity-alkalinity stress [20, 94]. Urea synthesis is an important strategy for ammonia detoxification in some fish [15]. Trioxypurine produced by arginine metabolism is the main source of urea synthesis in tilapia [95]. Under salinity-alkalinity stress, our results showed that tilapia fed a high-carbohydrate diet upregulated the arginine metabolism pathway compared with tilapia fed a high-protein diet, indicating that the high-carbohydrate diet was more conducive to urea metabolism and the ability to excrete ammonia. Research has also indicated that the urea cycle plays an important role in the response to alkalinity stress in tilapia [96]. In general, salinity-alkalinity stress can stimulate the energy metabolism of fish, and high-protein diets cause energy metabolism disorders in fish under salinity-alkalinity stress. However, high-carbohydrate feed not only is beneficial for providing energy to fish under salinity-alkalinity stress but can also promote the excretion of ammonia and maintain osmotic balance.

## Conclusions

Salinity-alkalinity stress damages the gill and liver structure of fish, causing oxidative stress, increasing energy requirements and the abundance of intestinal pathogens, and ultimately impeding growth and development. Although the high-protein diets do not impose the metabolic burden on the liver, they significantly increase the abundance of pathogenic bacteria in the gut and cause severe oxidative stress damage under salinity-alkalinity stress. Conversely, the high-carbohydrate diets not only effectively alleviated oxidative stress damage but also significantly enhanced the energy metabolism in tilapia, thereby markedly improving their ability to excrete ammonia and cope with osmotic challenges. In this study, the diet containing 27% protein and 35% carbohydrate was the most conducive to the growth and health of tilapia in saline-alkaline water.

## Data Availability

The datasets produced and/or analyzed during the current study are available from the corresponding author on reasonable request.

## Abbreviations

ASV: Amplicon sequence variants
BC: Blood cell
CAT: Catalase
CC: Chloride cell
CEV: Central veins
CF: Condition factor
*cs*: Citrate synthase
CV: Cytoplasmic vacuolation
DEGs: Differentially expressed genes
FCR: Feed conversion ratio
FW: Freshwater
*g6pase*: Glucose-6-phosphatase
GFC: Gill filament cartilage
GL: Gill lamella
*glut 2*: Glucose transporter 2
*gs*: Glycogen synthase
GSEA: Gene set enrichment analysis
GSH-PX: Glutathione peroxidase
*hk*: Hexokinase
HP: 42% Protein with 15% carbohydrate
HSI: Hepatosomatic index
*idh*: Isocitrate dehydrogenase
LP: 27% Protein with 35% carbohydrate
MDA: Malonaldehyde
MP: 35% Protein with 25% carbohydrate
MS-222: Tricaine methanesulfonate
N:C: Protein-to-carbohydrate
OEL: Outer epithelial layer
PA: Pancreatic acini
*p**epck*: Phosphoenolpyruvate carboxykinase
*pk*: Pyruvate kinase
ROS: Reactive oxygen species
SOD: Superoxide dismutase
SR: Survival rate
SW: Saline-alkaline water
WG: Weight gain rate
